# Psychiatric disorders of patients seeking obesity treatment

**DOI:** 10.1186/1471-244X-13-1

**Published:** 2013-01-02

**Authors:** Hung-Yen Lin, Chih-Kun Huang, Chi-Ming Tai, Hung-Yu Lin, Yu-Hsi Kao, Ching-Chung Tsai, Chin-Feng Hsuan, Su-Long Lee, Shu-Ching Chi, Yung-Chieh Yen

**Affiliations:** 1 Department of Psychiatry, E-Da Hospital, Yi-Da Road, Yan-Chau District, Kaohsiung, 824, Taiwan; 2 Bariatric & Metabolic International Surgery Center, E-Da Hospital, Kaohsiung, Taiwan; 3 Department of Internal Medicine, E-Da Hospital, Kaohsiung, Taiwan; 4 Department of Urology, E-Da Hospital, Kaohsiung, Taiwan; 5 Department of Endocrinology and Metabolism, E-Da Hospital , Kaohsiung, Taiwan; 6 Department of Pediatrics, E-Da Hospital, Kaohsiung, Taiwan; 7 Department of Obstetrics and Gynecology, E-Da Hospital, Kaohsiung, Taiwan; 8 Nursing Department, E-Da Hospital, I-Shou University, Kaohsiung, Taiwan; 9 School of Medicine, I-Shou University, Kaohsiung, Taiwan

**Keywords:** Obesity, Psychiatric disorders, Bariatric surgery

## Abstract

**Background:**

Obese and overweight people have a higher risk of both chronic physical illness and mental illness. Obesity is reported to be positively associated with psychiatric disorders, especially in people who seek obesity treatment. At the same time, obesity treatment may be influenced by psychological factors or personality characteristics. This study aimed to understand the prevalence of mental disorders among ethnic Chinese who sought obesity treatment.

**Methods:**

Subjects were retrospectively recruited from an obesity treatment center in Taiwan. The obesity treatments included bariatric surgery and non-surgery treatment. All subjects underwent a standardized clinical evaluation with two questionnaires and a psychiatric referral when needed. The psychiatric diagnosis was made thorough psychiatric clinic interviews using the SCID. A total of 841 patients were recruited. We compared the difference in psychiatric disorder prevalence between patients with surgical and non-surgical treatment.

**Results:**

Of the 841 patients, 42% had at least one psychiatric disorder. Mood disorders, anxiety disorders and eating disorders were the most prevalent categories of psychiatric disorders. Females had more mood disorders and eating disorders than males. The surgical group had more binge-eating disorder, adjustment disorder, and sleep disorders than the non-surgical group.

**Conclusion:**

A high prevalence of psychiatric disorders was found among ethnic Chinese seeking obesity treatment. This is consistent with study results in the US and Europe.

## Background

Obesity is becoming an important issue for health promotion. The World Health Organization estimated that around 1.5 billion adults were overweight (body mass index, BMI ≧ 25 kg/m^2^) and about 500 million people were obese (BMI ≧ 30 kg/m^2^) in 2008. In the United States (US), about 34% of people are obese [1]. Obese and overweight people have a higher risk of chronic physical illness, such as cardiovascular disease [2], stroke, diabetes mellitus, and hypertension [3]. The relationship between obesity and mental health is also considered important. In a community-based study, obesity was positively associated with several mental disorders, especially mood disorders and anxiety disorders [4]. Simon et al. [5] investigated 9125 representative samples in the US, and found that obesity was associated with significant increases in lifetime diagnoses of major depression, bipolar disorder, and panic disorder or agoraphobia. Scott et al. found that even after adjusting for sex and age, obese people still had a higher risk of mood disorder and anxiety disorder [6].

Different obesity treatments including diet control, behavior modification, pharmacotherapy, intra-gastric balloon and bariatric surgery have been used with obesity of different severities. Psychological factors may influence the effect of obesity treatment, no matter whether surgical or non-surgical. Obese people seeking treatment had more psychopathologies, such as anxiety, depression, eating pattern problems, and lower levels of self-esteem and quality of life than normal-weight controls [7,8]. Bariatric surgery is effective in weight loss and improvement of physical comorbidity in patients with morbid obesity [9]. Before bariatric surgery, a complete pre-operation survey by a multidisciplinary team, including psychological evaluation, is suggested by the NIH consensus [10].

A substantial proportion of patients receiving bariatric surgery has had psychiatric disorders, including anxiety disorder and mood disorder, throughout their life or even just before surgery [11,12]. The role of psychological factors in the outcome of bariatric surgery seems inconsistent. Some studies have found people with psychiatric disorders have less bodyweight loss [13], but others find no relationship or even more bodyweight loss [8]. An emphasis on psychological impact in the postoperative follow-up and detection of possible psychological needs or need of support throughout the treatment course are important [14].

Obesity has become a worldwide public health problem, including Asia. Asian populations have also been shown to have an elevated risk of type 2 diabetes, hypertension, and hyperlipidemia at a relatively low level of BMI, compared to Europeans. Therefore, the criteria for obesity and overweight are lower in Asia [15]. In Taiwan, obesity is defined as BMI ≧27 kg/m^2^ by the Department of Health. The estimated prevalence of obesity in Taiwan was 15.9%-19.2% among men and 10.7%-16.0% among women [16-18]. Most obesity studies in Asian countries focus more on the physical effects of obesity and less on mental aspects. In recent years, some studies in Asia have aimed to explore the psychological influence of obesity on community dwellers. Some studies were directed at people in weight reduction treatment, and most of them focused of their quality of life [19-21]. Psychiatric disorders are important in obesity treatment, but there is insufficient evidence on the prevalence of psychiatric disorders in people receiving obesity treatment in Asia.

Hence, we retrospectively evaluated the prevalence of mental disorders in people who sought obesity treatment in Taiwan. We hypothesized that psychiatric disorders were prevalent in obese people, similar to the situation in the US or Europe. We also hypothesized that there might be a difference in psychiatric disorder prevalence between patients undergoing bariatric surgery and those receiving non-surgical treatment.

## Methods

Subjects were recruited from an obesity treatment center in a university hospital in Taiwan. The obesity treatment center personnel comprised a multi-disciplinary team, and included a surgeon, internal physician, psychiatrist, urologist, obstetrics and gynecology doctor, nurse, case manager, dietician, and physical activity director. The obesity treatments in this center included non-surgical procedures: meal replacement, pharmacotherapy, psychiatric bio-feedback treatment and intra-gastric balloon, and surgery: bariatric surgery (sleeve, band, Roux-en-Y gastric bypass). First of all, the patients made up their mind as to the treatment modality. However, the patients who wanted to receive bariatric surgery had to meet the criteria of morbid obesity. They then needed to undergo a complete pre-operation evaluation, including a psychiatric evaluation. Our hospital has a committee in charge of determining whether the patients are eligible for bariatric surgery.

Patients received a complete physical evaluation during their first visit, and also completed two questionnaires: the Taiwanese Depression Questionnaire (TDQ) and the Chinese Health Questionnaire (CHQ). The TDQ is a 0-3-point, 18-question questionnaire used to screen clinical depressive disorder. [22]. The cut-off point in the community population is 18/19 points. The CHQ [23] is a 12-question, 2-reverse questions, 0-1-point questionnaire for screening “minor psychiatric disorders” such as anxiety disorder. The cut-off point in community surveys screening minor mental disorders is 4/5 points.

To avoid false negative results, we lowered the cut-off points for the CHQ and TDQ in our clinical practice. Those patients with CHQ <3 and TDQ <13 were regarded as having no psychiatric disorder. If any of the two scores were above the cut-off point (i.e., CHQ ≧3 or TDQ ≧13, or both), the patients would be referred to psychiatrists for further evaluation. The lifetime psychiatric diagnosis was made based on the psychiatrist’s diagnostic interview, using the Structured Clinical Interview for the DSM-IV (SCID).

We recruited all patients that visited the obesity treatment center of E-Da Hospital from January 2007 to December 2010. The exclusion criteria were age younger than 18 years, having incomplete BMI, TDQ or CHQ data, and refusal of psychiatric interview when needed.

All analyses were performed with the Statistical Package for Social Sciences, SPSS Version 17.0. The chi-square test was used to compare differences for categorical variables and the *t*-test was used to compare differences for continuous variables. The level of statistical significances was 0.05, two-tailed. Logistic regression was applied to examine whether BMI was associated with a psychiatric disorder.

This study was approved by the Institutional Review Board of E-Da Hospital, Taiwan (EMPR-098-073). The study design and performance complied with the Declaration of Helsinki.

## Results

**Figure 1:**
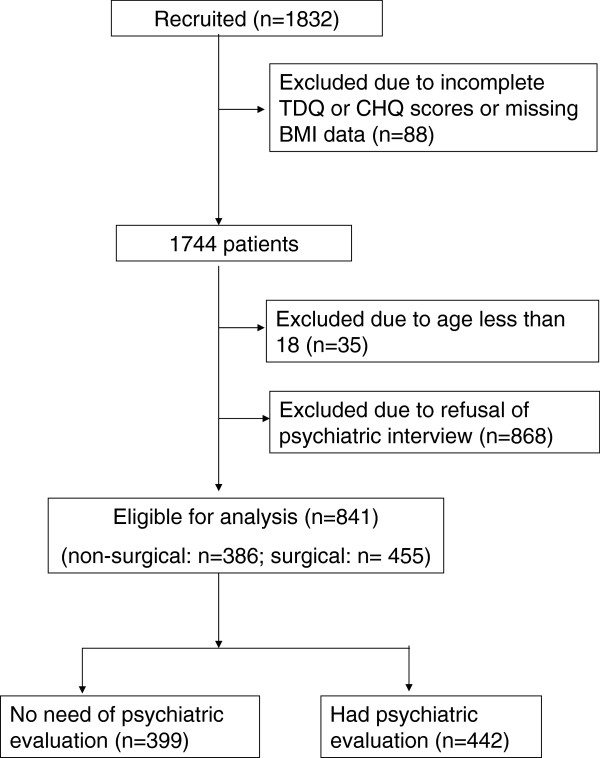
Flow of the study.

Of the 1832 subjects that were reviewed, the mean BMI was 35.2 kg/m^2^ (SD 8.9), 40.2% received bariatric surgery, 72.1% were female, and the mean age was 37.6 years (SD 11.73). Eighty-eight subjects were excluded due to incomplete TDQ and CHQ scores or missing BMI data; 35 were excluded because they were younger than 18 years; 868 were excluded because one of their questionnaire scores was higher than the cut-off point and they refused a further psychiatric interview. We included 841 patients in our analysis (Figure 1). Most of them were female (69.0%) and their mean age was 35.5 (SD 11.6) years. The mean BMI was 35.7 (SD 8.9) kg/m^2^. Of the recruited patients, 455 received bariatric surgery. The surgical group was younger, and had higher BMI, a higher TDQ score, and a higher educational level than the non-surgical group (Table 1). 

**Table 1:** Characteristics of patients seeking obesity treatment

	**Surgical**	**Non-surgical**	p*
	**(N=455)**	**(N=386)**	
**Female**	321 (70.5)	259 (67.1)	.281
**Age (years)(SD)**	34.1 (10.8)	37.2 (12.4)	<.001
**BMI at First Visit (SD)**	39.5 (8.3)	31.2 (7.3)	<.001
**Education, Years (%)**			
> 16	23 (5.1)	20 (4.8)	.028
13–16	234 (51.4)	195 (51.9)	
10–12	154 (33.8)	104 (27.4)	
7–9	26 (5.7)	29 (7.4)	
1–6	17 (3.7)	28 (7.2)	
0	1 (0.2)	5 (1.3)	
**Marital Status (%)**			
single	257 (56.5)	150 (39.2)	<.001
married	161 (35.4)	202 (52.7)	
divorced	30 (6.6)	24 (6.3)	
widowed	7 (1.5)	7 (1.8)	
**CHQ score (SD)**	4.2 (2.7)	3.8 (3.0)	.102
**TDQ score (SD)**	15.0 (11.6)	13.0(13.0)	.023

*Chi-square test was used for categorical variables; *t*-test was used for continuous variables.

Psychiatric diagnosis revealed that 42% of the patients had at least one psychiatric disorder. The 10 most prevalent psychiatric disorders were dysthymic disorder (20.5%), general anxiety disorder (17.2%), binge eating disorder (7.6%), major depressive disorder (7.2%), adjustment disorder (2.5%), sleep disorder (2.5%), bulimia nervosa (1.3%), bipolar disorder (1.2%), organic mental disorder (1.1%), and other mood disorders (1,1%). Mood disorders (27.1%), anxiety disorders (18.2) and eating disorders (8.6%) were the most prevalent categories of psychiatric disorders (Table 2). Women had more mood and eating disorders than men, but there was no sexual difference in anxiety disorders. 

**Table 2:** Prevalence of psychiatric disorders by sex

	**All**	**Male Female**		p*
	**(N=841)(%)**	**(N=261)(%)**	**(N=580)(%)**	
**Any psychiatric disorder**	353 (42.0)	84 (32.2)	269 (46.4)	<.001
**Mood disorders**				
Major depressive disorder	61 (7.3)	6 (2.3)	55 (9.5)	<.001
Dysthymic disorder	173 (20.6)	27 (10.3)	146 (25.2)	<.001
Depressive disorder, NOS	3 (0.4)	1 (0.4)	2 (0.3)	.931
Bipolar disorder	10 (1.2)	3 (1.1)	7 (1.2)	.943
Other mood disorder	9 (1.1)	1 (0.4)	8 (1.4)	.194
Any mood disorder	228 (27.1)	34 (13.0)	194 (33.4)	<.001
**Anxiety disorder**				
General anxiety disorder	145 (17.2)	45 (17.2)	100 (17.2)	>.999
OCD	1 (0.1)	1 (0.4)	0 (0)	.136
Panic disorder	6 (0.7)	2 (0.8)	4 (0.7)	.903
PTSD	3 (0.4)	1 (0.4)	2 (0.3)	.931
Specific phobia	1 (0.1)	0 (0)	1 (0.2)	.502
Social phobia	1 (0.1)	0 (0)	1 (0.2)	.502
Anxiety disorder, NOS	3 (0.4)	2 (0.8)	1 (0.2)	.181
Any anxiety disorder	153 (18.2)	47 (18.0)	106 (18.3)	.926
**Adjustment disorder**	21 (2.5)	7 (2.7)	14 (2.4)	.818
**Eating disorders**				
Bulimia nervosa	11 (1.3)	2 (0.8)	9 (1.6)	.354
Anorexia nervosa	2 (0.2)	0 (0)	2 (0.3)	.342
Binge eating disorder	64 (7.6)	7 (2.7)	57 (9.8)	<.001
Any eating disorder	72 (8.6)	8 (3.1)	64 (11.0)	<.001
**Psychotic disorders**				
Schizophrenia	7 (0.8)	1 (0.4)	6 (1.0)	.336
Other psychotic disorder	3 (0.4)	1 (0.4)	2 (0.3)	.931
**Substance use disorders**				
Alcohol-related disorders	5(0.6)	1 (0.4)	4 (0.7)	.593
Other substance-related disorder except for nicotine	0 (0)	0 (0)	0 (0)	
**Organic mental disorders**	9 (1.1)	3 (1.1)	6 (1/0)	.881
**Personality disorders**	3 (0.4)	0 (0)	3 (0.5)	.224
**Sleep disorders**	21 (2.5)	11 (4.2)	10 (1.7)	.032

*Chi-square test was used to compare differences in prevalence between males and females.

Surgical patients were more likely to have “other mood disorder” (1.8% versus 0.3%, p=0.035), binge eating disorder (10.3% versus 4.4%, p=0.001), adjustment disorder (3.7% versus 1.0%, p=0.012), and sleep disorders (3.7% versus 1.0%, p=0.012) than non-surgical patients (Table 3). Surgical patients still had a higher prevalence of overall psychiatric disorders, with borderline significance (54.1% versus 38.6%, p = 0.068). In terms of class of psychiatric disorders, surgery patients had more eating disorders than non-surgical patients, but there was no difference in any mood disorder (27.0% versus 27.2%, p=0.956) and any anxiety disorder (16.9% versus 19.7, p=0.300) between the two groups. 

**Table 3:** Prevalence of psychiatric disorders by intervention modalities

	**Surgical**	**Non- surgical**	p*
	**(N= 455)(%)**	**(N=386)(%)**	
**Any psychiatric disorder**	204 (54.1)	149 (38.6)	.068
**Mood disorders**			
Major depressive disorder	31 (6.8)	30 (7.8)	.593
Dysthymic disorder	97 (21.3)	76 (19.7)	.560
Depressive disorder, NOS	3 (0.7)	0	.110
Bipolar disorder	6 (1.3)	4 (1.0)	.707
Other mood disorder	8 (1.8)	1(0.3)	.035
Any mood disorder	123 (27.0)	105 (27.2)	.956
**Anxiety disorders**			
General anxiety disorder	72 (15.8)	73 (18.9)	.237
Obsessive-compulsive disorder	0	1 (0.3)	.277
Panic disorder	1 (0.1)	5 (0.6)	.065
Post-traumatic stress disorder	0	3 (0.8)	.060
Specific phobia	1(0.2)	0	.357
Social phobia	1 (0.2	0	.357
Anxiety disorder, NOS	3 (0.7)	0	.110
Any anxiety disorder	77 (16.9)	76 (19.7)	.300
**Adjustment disorders**	17 (3.7)	4 (1.0)	.012
**Eating disorders**			
Bulimia nervosa	5 (1.1)	6 (1.6)	.562
Anorexia nervosa	0	2 (0.2)	.127
Binge eating disorder	47 (10.3)	17 (4.4)	.001
Any eating disorder	49 (10.8)	23 (6.0)	.013
**Psychotic disorders**			
Schizophrenia	3 (0.7)	4 (1.0)	.549
Other psychotic disorder	3 (0.3)	0	.110
**Alcohol-related disorders**	2 (0.4)	3 (0.8)	.526
**Organic mental disorders**	4 (0.9)	5 (1.3)	.559
**Personality disorders**	0	3 (0.3)	.060
**Sleep disorders**	17 (3.7)	4 (1.0)	.012

*Chi-square test was used to compare differences in prevalence between surgical and non-surgical patients.

We examined whether BMI could predict the presence of psychiatric disorder using logistic regression. Univariate logistic regression showed that BMI could predict the presence of sleep disorder (OR 1.055 [95% CI 1.011-1.100], p=0.013), but that BMI was negatively associated with the presences of any anxiety disorder (OR 0.978 [95% CI 0.958-0.999], p=0.004) (Table 4). After adjusting for age, gender, education and marital status, BMI still could predict sleep disorder (OR 1.084 [95% CI 1.032-1.14], p=0.001), and was still negatively associated with the presence of any anxiety disorder (OR 0.975 [95% CI 0.954-0.997], p=0.027). Female gender was associated with any mood disorder (OR 3.155 [95% CI 2.094-4.752], p<0.001), any eating disorder (OR 3.728 [955 CI 1.734-8.016], p=0.001) and any psychiatric disorder (OR 1.779 [95% CI 1.292-2.448], p<0.001), adjusting for BMI, age, marital status, and educational level. 

**Table 4:** BMI predicting the presence of psychiatric disorders using logistic regression

	OR (95% CI)*	***p***	**Adjusted OR**	***p***
			**(95% CI)**	
**Any mood disorder**				
BMI	0.993 (0.976-1.010)	0.425	0.999 (0.980-1.018)	0.890
Female			3.155 (2.094-4.752)	<0.001
Age (years)			0.988 (9.68-1.008)	0.226
Marital status				
single				
married			1.188(0.765-1.846)	0.443
divorced or widowed			2.808 (1.508-5.230)	0.001
**Any anxiety disorder**				
BMI	0.978 (0.958-0.999)	0.04	0.975 (0.954-0.997)	0.027
Female			0.920 (0.619-1.385)	0.697
Age (years)			0.995 (0.973-1.018)	0.680
Marital status				
single				
married			0.853 (0.519-14.03)	0.531
divorced or widowed			0.871 (0.408-1.860)	0.722
**Any eating disorder**				
BMI	1.015 (0.988-1.042)	0.280	1.008 (0.980-1.038)	0.573
Female			3.728 (1.734-8.016)	0.001
Age (years)			0.973 (0.940-1.007)	0.114
Marital status				
single				
married			0.608 (0.0293-1.264)	0.183
divorced or widowed			1.152 (0.434-3.055)	0.776
**Sleep disorders**				
BMI	1.055 (1.011-1.100)	0.013	1.084(1.032-1.140)	0.001
Female			0.629 (0.247-1.599)	0.330
Age (years)			1.013 (0.957-1.072)	0.658
Marital status				
single				
married			4.653 (1.299-16.670)	0.018
divorced or widowed			1,676 (0.168-16.68)	0.659
**Any psychiatric disorder**				
BMI	1.003 (0.988-1.019)	0.6761.004	(0.987-10.02)	1.004
Female	1.779		(1.292-2.448)	<0.001
Age (years)			0.982 (0.965-1.000)	0.050
Marital status				
single				
married			1.209 (0.818-1.788)	0.340
divorced or widowed			2.152 (1.186-3.906)	0.012

*OR, odds ratio; CI, confidence interval.

Note. Educational level was also adjusted but not shown here. None of each educational level was predictive of the presence of psychiatric disorders.

## Discussion

This is, to our knowledge, the first paper in Asia to explore the prevalence of psychiatric disorders in Asian patients who seek obesity treatment. We retrospectively reviewed 841 patients who received different obesity treatments, including non-surgical procedures and bariatric surgery. The patients underwent a standardized clinical evaluation using two questionnaires, and psychiatric referral when needed. Clinical evaluation revealed that 42% of patients had at least one psychiatric disorder. Mood disorders and anxiety disorders were the most prevalent.

Kalarchian et al. [11] evaluated 288 bariatric surgery candidates. They found high prevalence rates of lifetime psychiatric disorders (66.3%) in these patients, even before surgery; 37.8% of patients had at least one psychiatric disorder. Rosenburger et al. [24] in the US and Muhlhans et al. [12] in Germany also found that patients had a high prevalence of psychiatric disorders before bariatric surgery (36.8%, and 72.6%, respectively). Our prevalence of any psychiatric disorder (54.1%) in the surgical group was lower than that of Kalarchian and Muhlhans, but higher than that of Rosenberger. Of all our patients receiving surgical or non-surgical treatment, the prevalence of any psychiatric disorder was 42%. This is not so different from the findings among Caucasians. We also found that mood disorders and anxiety disorders were the most prevalent classes of disorders, similar to the other studies. Eating disorders were the third most prevalent. We had a low frequency of substance abuse disorders (0.6%), and some patients had adjustment disorder and sleep disorders, which were not mentioned in the other three studies.

The differences in findings between our study and the other studies may be due to race, social factors and different study designs. The evaluation tool in the above three studies in the US and Germany was the structured interview, performed by well-trained psychologists. In our study, all psychiatric diagnoses were confirmed by board-certified psychiatrists. Our psychiatric evaluation is a part of the standardized pre-treatment evaluation process. However, patients might be worried about disclosing psychiatric problems if they thought it would affect their treatment, which may have lowered the prevalence of psychiatric disorders, as Muhlhans et al. [12] mentioned.

In our study, about 70% of patients were female. Females had a higher prevalence of mood disorders and eating disorders than males, but the males had more sleep disorders. There was no difference in anxiety disorder between the men and women. Similar outcomes were noted in our logistic regression models in Table 4. A previous community-based study, an international study in 13 areas worldwide involving 62,277 cases from the World Mental Health Survey [25], found that obesity increased the odds ratio of depression and anxiety, especially in females. The possible mechanism may be that women have more psychological stress from the stigma of obesity, greater dissatisfaction with their body image, and more eating problems [26].

Our finding is similar to that of Muhlhans’ study [12], in which women had more prevalent psychiatric disorders than men, but inconsistent with that of Kalarchian’s study. In Asia, the criteria for obesity are lower than in Europe and the US, which means that people are generally thinner in Asia. No matter whether physical or socio-cultural factors are involved, women in Asia whose BMI is the same as that of men may have more psychological stress when dealing with obesity. In our study, patients in the surgical group were younger and had higher BMI than those in the non-surgical group. Eating disorders, especially binge eating disorder, is prevalent in bariatric surgery patients [11] and has affected the outcome of weight loss after surgery [27,28]. Postoperative binge eating disorder can predict a poor surgical outcome. However, in our study, there were no differences between the two groups in terms of the prevalence of the other two important psychiatric disorders: mood and anxiety disorders. These two disorders may be affected by many different and complex psychosocial factors, not only BMI. Patients in the surgical group had a higher prevalence of several specific psychiatric disorders (adjustment disorder, binge eating disorder, and sleep disorders) than their non-surgical counterparts, but overall, psychiatric disorders were prevalent in both groups. This implies that people who seek obesity treatment, no matter the treatment they receive, suffer from similar psychopathological processes, with some exceptions.

The mood disorders, including bipolar disorder and depressive disorder, were the most prevalent class of disorders in our study. Patients with bipolar disorder are at a higher risk of being overweight and obese. The possible risk factors for weight gain in bipolar disorder patients include comorbid binge-eating disorder; the number of depressive episodes, treatment with medications associated with weight gain, low rates of exercise [29], age, comorbid anxiety disorders, duration of depressive episodes, and history of hospitalization for depression [30]. The interventions used with obese bipolar patients should include better metabolite profile medication, adjunctive pharmacotherapy for weight loss, and the integration of lifestyle factors and weight-management counseling in the long-term care plan [30,31]. Depression may lower the patient’s level of energy and motivation, or change their appetite, making them less careful about their health. The relationship between depression and obesity is bi-directional, as seen in the meta-analytical evidence. Researchers found that obese persons had a 55% increased risk of developing depression over time, whereas depressed persons had a 58% increased risk of becoming obese (26). The possible etiology of the association between obesity and depression may be biological and psychological, but this requires further evaluation.

In this study, we found that higher BMI was associated with sleep disorders. Sleep disturbance in obese people may be related to anatomical factors, endocrinological factors, and metabolic circadian abnormalities of the physical condition [32]. About 70% of people with obstructive sleep apnea are obese. On the other hand, the prevalence of sleep apnea disorder among obese people is approximately 40% [33]. In previous research, attention deficit hyperactivity disorder (ADHD) was reported to be associated with obesity, binge eating behavior and sleep/alertness problems [34]. Cortese et al. proposed that obesity might be one of the factors associated with the sleep/alertness problem and manifest as ADHD-like symptoms. The association of obesity with ADHD is a novel area in need of attention and further study.

Our study had an adequate sample size (841 subjects, including 455 surgical patients and 368 non-surgical patients). We compared the characteristics and clinical correlates of psychiatric disorders in obese patients. Few studies have explored the differences before. The psychiatric disorders in this study were diagnosed by board-certified psychiatrists and hence had good reliability. However, some limitations of our study should be noted. The high rates of refusal of psychiatric evaluation may affect our results. One of the explanations may be that people in Taiwan do not understand that obesity is not only a physical disease, but also a possible mental disorder. They may worry about the stigma and are unwilling to visit a psychiatrist. All patients in our study were recruited from one university hospital. The community hospitals in Taiwan do not provide bariatric surgery, though some community hospitals treat obese people with non-surgical interventions. No known demographic difference between patients in community hospitals and university hospitals has ever been reported.

In recent years, many studies have focused not only on pre-treatment psychiatric problems, but also on post-treatment follow-up. Obesity treatment, including bariatric surgery, is still under development in Asia, and psychiatric involvement is imperative for a comprehensive treatment. Future studies should focus on the effects of ethnicity and culture, which are diverse in Asian countries.

## Conclusions

A high prevalence of psychiatric disorders was found among ethnic Chinese seeking obesity treatment. Mood disorders, anxiety disorders and eating disorders were the most prevalent categories of psychiatric disorders. The surgical group had more binge-eating disorders, other mood disorders, adjustment disorder, and sleep disorders than the non-surgical group. Psychiatric evaluation might be an important factor in complete obesity treatment and require further study.

## Competing interests

The authors declare no competing interests.

## Authors’ contributions

LH-Y and YY-C were responsible for the study design, data collection, and manuscript writing. HC-K, TC-M and LH-Y recruited participants and collected data. TC-C and HC-F analyzed data. KY-H, LS-L and CS-C helped analyze data and prepare the manuscript. All authors read and approved the final manuscript.

## Pre-publication history

The pre-publication history for this paper can be accessed here:

http://www.biomedcentral.com/1471-244X/13/1/prepub

## References

[B1] Flegal KM Carroll MD Ogden CL Curtin LR Prevalence and trends in obesity among US adults, 1999–2008 JAMA 2010 303 235 241 10.1001/jama.2009.2014 20071471

[B2] Ebbeling CB Pawlak DB Ludwig DS Childhood obesity: public-health crisis, common sense cure Lancet 2002 360 473 482 10.1016/S0140-6736(02)09678-2 12241736

[B3] Must A Spadano J Coakley EH Field AE Colditz G Dietz WH The disease burden associated with overweight and obesity JAMA 1999 282 1523 1529 10.1001/jama.282.16.1523 10546691

[B4] Stunkard AJ Faith MS Allison KC Depression and obesity Biol Psychiatry 2003 54 330 337 10.1016/S0006-3223(03)00608-5 12893108

[B5] Simon GE Von Korff M Saunders K Miglioretti DL Crane PK van Belle G Kessler RC Association between obesity and psychiatric disorders in the US adult population Arch Gen Psychiatry 2006 63 824 830 10.1001/archpsyc.63.7.824 16818872 PMC1913935

[B6] Scott KM McGee MA Wells JE Oakley Browne MA Obesity and mental disorders in the adult general population J Psychosom Res 2008 64 97 105 10.1016/j.jpsychores.2007.09.006 18158005

[B7] Abiles V Rodriguez-Ruiz S Abiles J Mellado C Garcia A Perez de la Cruz A Fernandez-Santaella MC Psychological characteristics of morbidly obese candidates for bariatric surgery Obes Surg 2010 20 161 167 10.1007/s11695-008-9726-1 18958537

[B8] Averbukh Y Heshka S El-Shoreya H Flancbaum L Geliebter A Kamel S Pi-Sunyer FX Laferrere B Depression score predicts weight loss following roux-en-Y gastric bypass Obes Surg 2003 13 833 836 10.1381/096089203322618605 14738665

[B9] Buchwald H Avidor Y Braunwald E Jensen MD Pories W Fahrbach K Schoelles K Bariatric surgery: a systematic review and meta-analysis JAMA 2004 292 1724 1737 10.1001/jama.292.14.1724 15479938

[B10] NIH conference Gastrointestinal surgery for severe obesity. Consensus development conference panel Ann Intern Med 1991 115 956 961 1952493

[B11] Kalarchian MA Marcus MD Levine MD Courcoulas AP Pilkonis PA Ringham RM Soulakova JN Weissfeld LA Rofey DL Psychiatric disorders among bariatric surgery candidates: relationship to obesity and functional health status Am J Psychiatry 2007 164 328 334 quiz 374. 10.1176/appi.ajp.164.2.328 17267797

[B12] Muhlhans B Horbach T de Zwaan M Psychiatric disorders in bariatric surgery candidates: a review of the literature and results of a German prebariatric surgery sample Gen Hosp Psychiatry 2009 31 414 421 10.1016/j.genhosppsych.2009.05.004 19703634

[B13] Kinzl JF Schrattenecker M Traweger C Mattesich M Fiala M Biebl W Psychosocial predictors of weight loss after bariatric surgery Obes Surg 2006 16 1609 1614 10.1381/096089206779319301 17217637

[B14] Pull CB Current psychological assessment practices in obesity surgery programs: what to assess and why Curr Opin Psychiatry 2010 23 30 36 10.1097/YCO.0b013e328334c817 19926994

[B15] WHO expert consultation Appropriate body-mass index for Asian populations and its implications for policy and intervention strategies Lancet 2004 363 157 163 14726171 10.1016/S0140-6736(03)15268-3

[B16] Shimokawa S Chang HH Pinstrup-Andersen P Understanding the differences in obesity among working adults between Taiwan and china Asia Pac J Clin Nutr 2009 18 88 95 19329401

[B17] Chu NF Prevalence of obesity in Taiwan Obes Rev 2005 6 271 274 10.1111/j.1467-789X.2005.00175.x 16246212

[B18] Hwang LC Bai CH Chen CJ Prevalence of obesity and metabolic syndrome in Taiwan J Formos Med Assoc 2006 105 626 635 10.1016/S0929-6646(09)60161-3 16935763

[B19] Chang CY Hung CK Chang YY Tai CM Lin JT Wang JD Health-related quality of life in adult patients with morbid obesity coming for bariatric surgery Obes Surg 2010 20 1121 1127 10.1007/s11695-008-9513-z 18463932 PMC2910893

[B20] Loh CB Chan YH Psychological symptoms in people presenting for weight management Ann Acad Med Singapore 2010 39 778 782 21063638

[B21] Kim JY Oh DJ Yoon TY Choi JM Choe BK The impacts of obesity on psychological well-being: a cross-sectional study about depressive mood and quality of life J Prev Med Public Health 2007 40 191 195 10.3961/jpmph.2007.40.2.191 17426433

[B22] Lee Y Yang MJ Lai TJ Chiu NM Chau TT Development of the Taiwanese depression questionnaire Chang Gung Med J 2000 23 688 694 11190378

[B23] Cheng TA Williams P The design and development of a screening questionnaire (CHQ) for use in community studies of mental disorders in Taiwan Psychol Med 1986 16 415 422 10.1017/S0033291700009247 3726013

[B24] Rosenberger PH Henderson KE Grilo CM Psychiatric disorder comorbidity and association with eating disorders in bariatric surgery patients: a cross-sectional study using structured interview-based diagnosis J Clin Psychiatry 2006 67 1080 1085 10.4088/JCP.v67n0710 16889451

[B25] Scott KM Bruffaerts R Simon GE Alonso J Angermeyer M de Girolamo G Demyttenaere K Gasquet I Haro JM Karam E Obesity and mental disorders in the general population: results from the world mental health surveys Int J Obes (Lond) 2008 32 192 200 10.1038/sj.ijo.0803701 17712309 PMC2736857

[B26] Striegel-Moore RH Silberstein LR Rodin J Toward an understanding of risk factors for bulimia Am Psychol 1986 41 246 263 3457546 10.1037//0003-066x.41.3.246

[B27] Busetto L Segato G De Luca M De Marchi F Foletto M Vianello M Valeri M Favretti F Enzi G Weight loss and postoperative complications in morbidly obese patients with binge eating disorder treated by laparoscopic adjustable gastric banding Obes Surg 2005 15 195 201 10.1381/0960892053268327 15802061

[B28] Scholtz S Bidlake L Morgan J Fiennes A El-Etar A Lacey JH McCluskey S Long-term outcomes following laparoscopic adjustable gastric banding: postoperative psychological sequelae predict outcome at 5-year follow-up Obes Surg 2007 17 1220 1225 10.1007/s11695-007-9212-1 18074498

[B29] Keck PE McElroy SL Bipolar disorder, obesity, and pharmacotherapy-associated weight gain J Clin Psychiatry 2003 64 1426 1435 10.4088/JCP.v64n1205 14728103

[B30] Goldstein BI Liu SM Zivkovic N Schaffer A Chien LC Blanco C The burden of obesity among adults with bipolar disorder in the united states Bipolar Disord 2011 13 387 395 10.1111/j.1399-5618.2011.00932.x 21843278 PMC3157038

[B31] McElroy SL Keck PE Jr Obesity in bipolar disorder: an overview Curr Psychiatry Rep 2012 14 650 658 10.1007/s11920-012-0313-8 22903246

[B32] Akinnusi ME Saliba R Porhomayon J El-Solh AA Sleep disorders in morbid obesity Eur J Intern Med 2012 23 219 226 10.1016/j.ejim.2011.10.016 22385877

[B33] Resta O Foschino-Barbaro MP Legari G Talamo S Bonfitto P Palumbo A Minenna A Giorgino R De Pergola G Sleep-related breathing disorders, loud snoring and excessive daytime sleepiness in obese subjects Int J Obes Relat Metab Disord 2001 25 669 675 10.1038/sj.ijo.0801603 11360149

[B34] Cortese S Morcillo Penalver C Comorbidity between ADHD and obesity: exploring shared mechanisms and clinical implications Postgrad Med 2010 122 88 96 10.3810/pgm.2010.09.2205 20861592

